# “Furry tales”: pet ownership’s influence on subjective well-being during Covid-19 times

**DOI:** 10.1007/s11135-021-01303-7

**Published:** 2021-12-22

**Authors:** Svenja Damberg, Lena Frömbling

**Affiliations:** Institute of Human Resource Management and Organizations (W-9), Hamburg University of Technology (TUHH), Am Schwarzenberg-Campus 4(D), 21073 Hamburg, Germany

**Keywords:** Subjective well-being, Human-pet relationship, Need support, Covid-19, PLS-SEM

## Abstract

The social distancing required during Covid-19 times tended to make people feel lonelier than usual. Those with pets might, however, have experienced this less, because pets are known for fostering their owners’ subjective well-being. Building on a recently published structural equation model, our study enhances the understanding of subjective well-being by including the construct social distancing during Covid-19 times. In order to answer our research question—*How does human-pet relationship need support influence subjective well-being by considering social isolation during Covid-19 times?*—we build on the basic needs theory, assuming that humans as well as their pets have an inherent need of autonomy, relatedness, and competence. Using a multivariate data analysis method, namely partial least squares structural equation modeling (PLS-SEM), we establish a path model and examine the relationship between human-pet relationship need support and subjective well-being by including psychological distress and social isolation during Covid-19 times as mediators. We operationalize subjective well-being as a three-dimensional construct consisting of positive affect, happiness, and life satisfaction. In a sample of 215 pet owners in the USA, supporting their need increases subjective well-being, and decreases the psychological distress and loneliness caused by social isolation during Covid-19 times. Furthermore, psychological distress decreases subjective well-being, whereas perceived loneliness during Covid-19 times does not. Our main contributions are to not only enhance our knowledge on the importance of human-pet relationships in critical times, but also to provide policy makers with insights into what influences people’s subjective well-being, which is closely related to their psychological health.

## Introduction

The Covid-19 pandemic has forced people around the globe to cope with social isolation (Mayers 2021), which could have negative psychological consequences for them (Casali et al. 2021). One of governments’ strategies for coping with the pandemic was to encourage their citizens to maintain a social distance, or even self-quarantine, to prevent the virus from spreading (Banerjee and Rai 2020; Lu et al. 2021; Van Bavel et al. 2020). This social isolation measure might have led to feelings of loneliness and boredom, which, in turn, could have long-term health consequences and negatively impacted individuals’ well-being (Van Bavel et al. 2020; Banerjee and Rai 2020). Even pandemic-independent, studies have shown that greater isolation leads to a deterioration of perceived health in individuals (Sironi and Wolff 2021).

In Western societies, well-being is mainly experienced in close circles of family and friends (Delle Fave et al. 2011, 2016), but even minimal social interactions with strangers in individuals’ everyday life can contribute to their subjective well-being (Gunaydin et al. 2021). Moreover, well-being has become an ever more important topic in today’s society, since long-term feelings of unhappiness are associated with immense healthcare costs. Worldwide, mental, neurological and substance use disorders are estimated to lead to annual economic output losses of $2.5–8.5 trillion, with these numbers expected to double by 2030 (Batadarené and Solano 2019). Even though the knowledge about quarantining’s effect on mental health is still scarce (Lu et al. 2021), a recent study by Casali et al. (2021) has indicated that Covid-19’s psychological impact is likely to persist well into the future.

However, prior research has also found that pet ownership can be positively related to human health and health behaviors (Utz 2014), while social isolation has strengthened the bond between humans and dogs in times of the worldwide pandemic (Mayers 2021). In fact, the latest report by the American Pet Products Association stated an increase in new pet adoptions during the Covid-19 pandemic in the USA in 2020 (APPA 2021). Since animals play an important role in several aspects of human psychological and cultural life (Herzog 2011), the relationship between pet ownership and well-being has lately received a great deal of research attention (Kanat-Maymon et al. 2016, 2021; Bao and Schreer 2016; Amiot and Bastian 2015). Nevertheless, empirical research on the human-animal bond is still scarce (Amiot et al. 2020), encouraging a strong call to develop this research area further (Amiot and Bastian 2015; Herzog 2011).

Research findings suggest that pets, such as dogs, do have a positive influence on their owners’ well-being and health (Headey et al. 2008), since it is indicated that dog and cat owners need fewer doctoral visits and report less heart issues and sleeping disorders (Headey 1999). However, there is no clear indication whether the pet effect on humans is positive or negative, or whether there is any effect at all (Herzog 2011), since research has shown conflicting findings (Fraser et al. 2020). Similar, research on the benefits of subjective well-being through improved social relationships, among many other causes, does not receive enough attention (Maccagnan et al. 2019). In this regard, there is a strong need for research on the Covid-19 lockdown’s specific impact and on the ripple effect this crisis could have once the situation returns to normal (Oliva and Johnston 2021).

Our study was conducted during the worldwide Covid-19 pandemic, since we aimed at providing an answer to help solving the larger puzzle of this current pandemic’s consequences. We follow recent calls by integrating two important research agendas – namely, research on the human-pet relationship and research on Covid-19. Our study contributes to the current research agenda on need support’s effect, that is satisfying a human’s inherent needs, on subjective well-being by building on and adapting a structural equation model (SEM) as suggested by Kanat-Maymon et al. (2021). Furthermore, we include a mediation analysis to further study the impact of quarantine on mental health, as called on by Lu et al. (2021). Our research questions is: *How does human-pet relationship need support influence subjective well-being by considering social isolation during Covid-19 times?* By answering this question, we further develop the research agenda on human-pet relationships and subjective well-being, taking into consideration the special circumstances during social isolation.

We collected a sample of pet owners through Amazon Mechanical Turk (MTurk), and evaluated our model by means of partial least squares structural equation modeling (PLS-SEM). Our results show that human-pet relationship need support increases subjective well-being and decreases psychological distress as well as loneliness caused by social isolation during Covid-19 times. Furthermore, as hypothesized, psychological distress decreases subjective well-being, although, surprisingly, loneliness during Covid-19 does not. In light of the above, our main contributions are threefold: (1) We enhance the understanding of subjective well-being, (2) extend research on the human-pet relationship, and (3) provide an answer to the ongoing discussion on mental health during crises, such as the current Covid-19 pandemic. Consequently, we provide a model for researchers to develop our fellow understanding of the human-pet relationship, for pet owners to better understand their relationship with their pets, and for policy makers to improve citizen’s psychological health. Since it is unclear how long the Covid-19 pandemic and its consequences will remain a global matter of concern, health care systems could build on our core finding that owning a pet – especially in times of social isolation – can indeed be beneficial for humans.

We structure this study as follows: First, we explore the human-pet relationships’ theoretical underpinnings (Sect. 2) develope our theoretical model’s components and deduce the underlying hypotheses in Sect. 3. In Sect. 4, we introduce our constructs’ operationalizations, sample, and methodological approach. We assess our results in Sect. 5 and discuss them in light of theoretical and practical implications (Sect. 6). In the last section, we specify our study’s limitations and potentials for future research.

## Theoretical background

Animals are part of a variety of human life’s contexts, including, for example, leisure, work, and health (Amiot and Bastian 2015). Moreover, human-animal relationships have implications for both humans and animals (Amiot and Bastian 2015) in which the positive impact from pets on humans is called the pet-effect (Janssens et al. 2020). Since we cannot as yet interpret an animal’s feelings with certainty, research often draws on the anthropomorphism discipline, which maintains that pet owners ascribe human-like characteristics (Amiot and Bastian 2015; Brown et al. 2016) and needs (Kanat-Maymon et al. 2021) to their pets. They thereby construct a perceived relationship with them (Archer 1997) as well as invest time, energy, and money in their pets (Kanat-Maymon et al. 2016).

At the same time, pet need satisfaction might be an independent source of need satisfaction instead of being a projection of human-human relationships onto human-pet relationships (Kanat-Maymon et al. 2016). A human-pet relationship involves, to a certain extent, mutual interdependence (Kanat-Maymon et al. 2016). Under certain circumstances, pets might even replace and act as a substitute for human-human friendships (Dotson and Hyatt 2008). This tends to occur more often when people have fewer or less fulfilling human relationships, since pets provide them with the unconditional love they do not otherwise experience (Archer 1997).

## Model development and hypotheses

### Theoretical model

**Fig. 1:**
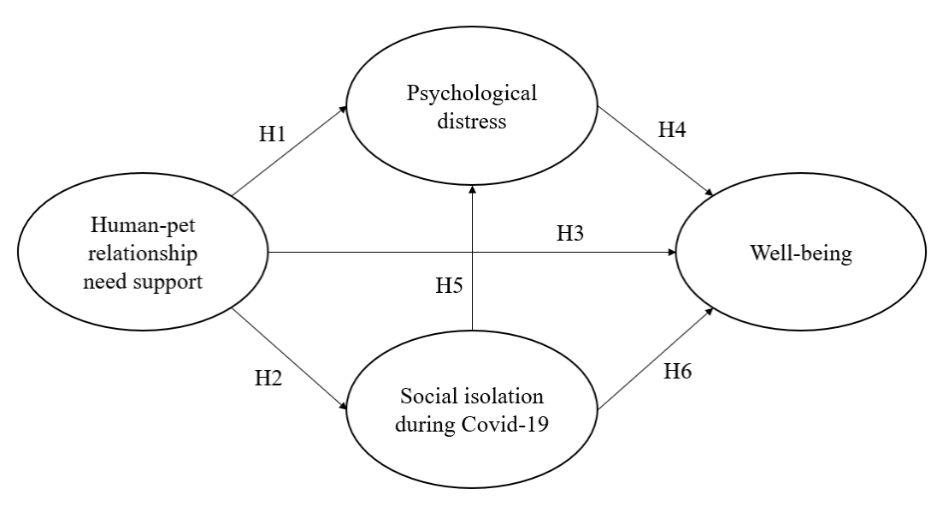
**Theoretical model** Source: own illustration based on Kanat-Maymon et al. (2016) and Kanat-Maymon et al. (2021).

In our model, we build on the relationships described in the studies by Kanat-Maymon et al. (2016) and Kanat-Maymon et al. (2021). The latter study finds that giving dogs need support contributes to their owners’ well-being, lessens their psychological distress, and leads to greater feelings of closeness to their dogs. However, in our model (Fig. 1), we set subjective well-being as the single target construct. Furthermore, we exclude closeness to the dog for various reasons (for further explanations, see the discussion section). In our model, human-pet relationship need support is measured as a higher-order construct (HOC) comprising a giving and a receiving dimension of need support. Social isolation during Covid-19 and psychological distress are considered to be individual and sequential mediators in the relationship between human-pet relationship need support and subjective well-being.

### Subjective well-being

By today, we know that gross domestic product (GDP) per capita provides useful insights into well-being, but is not sufficient to capture other important domains, such as social relations (Ivaldi et al. 2016), hence, there is a tendency in research to move beyond such a measurement (Maccagnan et al. 2019) to other constructs, such as subjective well-being, since it complements research on quality of life (Sirgy et al. 2006; MacLeod 2015)and adopts a variety of different views, traditions, and definitions of well-being. These views of well-being vary regarding the extent to which they integrate people’s subjective states when evaluating well-being, as opposed to people having an objective check-list, which, by completing it, results in a person’s well-being. The least constrained subjective state is the one in which a person judges his or her well-being in terms of happiness. Objective views assign ideal states to a person’s life, regardless of this person’s evaluation of subjective happiness (MacLeod 2015). In this study, we focus on the perception of one’s well-being, which we therefore refer to as subjective well-being.

Within well-being research, two main research traditions deal with the dimensions of well-being (Demir and Özdemir 2010). The first tradition, referred to as hedonic or subjective well-being, focuses on pleasure and happiness, while the second tradition, called eudaimonic or psychological well-being, focuses on meaning and self-realization (Ryan and Deci 2001). The attainment of eudaimonic well-being is a double-edged sword, as it might be achieved either in the short- or long-term, but not necessarily both. For example, drug misuse evokes good feelings in the short-term but is damaging health in the long-term, or a person might sacrifice short-term well-being in exchange for long-term goals (Delle Fave and Bassi 2009). As multi-facetted research on subjective well-being is, so are its definitions, while generally including both cognitive as well as affective components (Sirgy et al. 2006). In our study, we consider subjective well-being as an overall concept of happiness, life satisfaction, and positive affect (Diener 1984).

*Happiness* is subjective well-being’s first dimension, associated with mental health, and, furthermore, an important outcome of quality of life (Burns and Crisp 2021). In comparison to human happiness, animal happiness might only be based on happiness’s affective component, while human happiness, in addition to the affective experience, also includes a cognitive comparison of the elements under consideration (Acosta-González and Marcenaro-Gutiérrez 2021; Webb et al. 2019). Humans mostly associate happiness with health and relationships (Bojanowska and Zalewska 2016), while happiness itself can also be a predictor of self-reported health (Acosta-González and Marcenaro-Gutiérrez 2021).

*Life satisfaction* is the cognitive aspect of happiness (Diener 1984) and the second facet of subjective well-being in our model. Bao and Schreer (2016) found that pet owners are more satisfied with their lives than non-owners. A possible explanation for this difference can be found by distinguishing between cognition and affect. Cognition, which is relatively more resistant to certain circumstances, is activated when people evaluate their overall life satisfaction, also taking their human-pet relationship into account.

*Positive affect* is the third dimension of our subjective well-being construct. Affect includes facial, physiological, motivational, behavioral, and cognitive components (Diener 1994). Positive affect means having an enthusiastic, active, and alert feeling, while negative affect deals with subjective distress and unpleasurable engagement. These two states can be regarded as distinctive dimensions and not necessarily as two poles of one scale (Watson et al. 1988). Furthermore, Janssens et al. (2020) found interaction with companion animals to have an impact on positive affect.

### Human-pet relationship need support

Need support is closely linked to the self-determination theory (SDT). Basic psychological needs are defined as “innate psychological nutriments that are essential for ongoing psychological growth, integrity, and well-being” (Deci and Ryan 2000, p. 229). According to the SDT, three basic needs, namely autonomy, competence, and relatedness, specify the conditions required for psychological growth, integrity, and well-being. These three needs are thus universal and fundamental human needs.

The need for *autonomy* is linked to experiencing volition and endorsing of one’s actions (Kanat-Maymon et al. 2021). This need might be frustrated when, for example, one’s thoughts, behaviors, or feelings are pressurizing, alienating, or conflicting (Rodríguez-Meirinhos et al. 2020). The need for *competence* includes a desire to feel capable and accomplished (Kanat-Maymon et al. 2021), in the sense of effectiveness regarding pursuing one’s goals, developing one’s skills, and coping with daily challenges (Rodríguez-Meirinhos et al. 2020). This need is frustrated when someone fails to accomplish daily tasks and achieve desired outcomes, or does so inadequately (Rodríguez-Meirinhos et al. 2020). The need for *relatedness* regulates a desire to feel connected to and understood by others (Kanat-Maymon et al. 2021). This need is frustrated when people feel socially rejected or disconnected from others (Rodríguez-Meirinhos et al. 2020).

Kanat-Maymon et al. (2016) found that dogs’ ability to love unconditionally increases humans’ feelings of self-worth and competence. When a person anthropomorphizes and thereby ascribes certain human needs to their dog, supporting these needs is also beneficial for the dog owner (Kanat-Maymon et al. 2021). In this way, the care that dog owners give to their dogs in order to provide them with decent living conditions and to communicate a feeling of warmth, also supports these owners’ relatedness need. Similarly, dog owners provide a feeling of autonomy through activities that their dogs enjoy with respect to their personalities (Kanat-Maymon et al. 2021). When successfully teaching their pets behavioral regulations, this might also fulfill the dog owners’ competence need (Kanat-Maymon et al. 2021).

By combining the SDT with the conservation of resources theory (COR) by Hobfoll (1989), we can further explain the influence from human-pet relationship need support onto psychological distress as well as social isolation. According to the COR, resource loss produces stress, while resource gain receives more importance in a situation of resource loss (Hobfoll 2001; Wells et al. 1999). Benight et al. (1999) further state that lost resources contribute to explaining psychological reactivity following a natural disaster. Since our study was conducted during the ongoing Covid-19 pandemic, we can only extrapolate and relate this finding to this current crisis. Moreover, Yu et al. (2021) declare that Covid-19 is an initiator of several resource losses, social resources being one of them. People who inherit a resource that is able to substitute the missing social resources might be able to better cope with psychological distress and social isolation. In this sense, we consider the healthy relationship between a human and their pet as a resource that fills the void of social interaction. Consequently, we hypothesize the following:

*H1: Human-pet relationship need support decreases psychological distress.*

*H2: Human-pet relationship need support decreases feelings of social isolation due to Covid-19.*

*H3: Human-pet relationship need support increases subjective well-being.*

### Psychological distress

Psychological distress refers to a negative feeling about one’s life. Research distinguishes between the simple absence of happiness and more comprehensive measures of the concept, such as including levels of nervousness, agitation, fatigue, and negative affect (Meng and D’Arcy 2016). Previous studies found that animals can protect people with lower social support levels against negative effects of stressful situations’ negative effects (Amiot and Bastian 2015), in that the presence of a companion animal buffers against negative feelings (Janssens et al. 2020). Furthermore, pet ownership and social support are found to be predictors of survival in and after crisis situations, including illnesses (Friedmann and Thomas 1995), or posttraumatic growth (Dominick et al. 2020). However, having to care for a pet might not in itself lead to lower levels of depression (Miltiades and Shearer 2011). Another study found that pets have a positive influence on their owners’ overall health (Headey et al. 2008). Nevertheless, studies have shown that distress reduces people’s overall psychological well-being (Meng and D’Arcy 2016). We therefore hypothesize the following:

*H4: Psychological distress decreases subjective well-being.*

### Social isolation and loneliness

Social isolation can be defined “as a lack of personal contacts with peers and an absence of profound relationships” (Sironi and Wolff 2021, p. 2099), while loneliness is often described as a state of being without company or being isolated from a community or society (Banerjee and Rai 2020). Feelings of loneliness are not synonymous with being alone, but involve feelings of isolation, disconnectedness, and not belonging (Hughes et al. 2004). A discrepancy arises when the desired and the experienced amount of closeness through social relationships are conflicting (Mund and Johnson 2021). Prior research found that social need fulfillment is stable across age groups, since the importance of specific social needs’ fulfillment to experience happiness does not differ along the life span (Buijs et al. 2021), similar to the general experience of personal well-being (Cummins et al. 2003). Consequently, the impact of a lockdown on loneliness affects people of all ages, rather than just a particular age group (Oliva and Johnston 2021).

Social support systems, which could refer to friends, family, co-workers, but also pets, contribute to feelings of well-being, thereby addressing the current global loneliness challenge (Odekerken-Schröder et al. 2020). Oliva and Johnston (2021) found that dog ownership helps protecting people against loneliness, in that the pet acts as a companion, therefore helping them endure the lockdown experience. An important aspect in a socially isolated situations is to have a physical connection with a companion, that is feeling and touching another living being (Oliva and Johnston 2021) and an enhanced mood due to interaction with for example, a dog (Powell et al. 2019). This positive impact might occur relatively fast after acquiring a dog (Powell et al. 2019). This phenomenon also seems to hold the other way around–dogs and cats are positively affected, since they receive more attention during Covid-19 times (Oliva and Johnston 2021). Kalenkoski and Korankye (2021) found that the benefits of walking, exercising, or playing with pets are higher for older people who live alone, being, thereby, more important than nonpet-related activities. We therefore hypothesize that:

*H5: Social isolation due to Covid-19 increases psychological distress.*

*H6: Social isolation due to Covid-19 decreases subjective well-being.*

## Method and data

### Operationalization

All our constructs’ conceptualizations had already been tested and validated in previous studies (Table 1). We based the majority of our measurement scales on those of Kanat-Maymon et al. (2021), which offer a good basis for validity and reliability. We adapted these scales from their focus on dogs to a focus on pets in general. Since we aimed to capture a complex social phenomenon, namely subjective well-being, we also needed to capture its different perspectives (Lauro et al. 2018). In order to do that, we adjusted the well-being scale according to the Diener (1984) conceptualization, in which well-being consists of three facets, namely life satisfaction, happiness, and positive affect. Dyer (2014) developed a five-item psychometric scale to measure distress. In line with Kanat-Maymon et al. (2021), we used three of the original scale’s items, eliminating two extreme items on suicidal tendencies. Affect is operationalized by an overall positive feeling about one’s life. Our mediator, namely social isolation during Covid-19 times, is based on the study by Oliva and Johnston (2021), who used a short version of the University of California, Los Angeles (UCLA) loneliness scale (Hughes et al. 2004). We measured most of our constructs on a Likert scale ranging from 1 (strongly disagree) to 5 (strongly agree). Perceived social isolation in Covid-19 times was an exception and we used a Likert scale ranging from 1 (never) to 4 (often). All constructs were operationalized in a reflective manner. We measured human-pet relationship need support as a HOC, comprising three items for both giving and receiving need support as lower order constructs (LOCs). We estimated the HOC by using the two-stage approach.

**Table 1:** Measurement and operationalization

Construct	Items	Scale	Sources
Daily giving of need support	When I interact with my pet, I try to show it that I really care for it. When I interact with my pet, I try to let it feel competent. When I interact with my pet, I try to let it feel free to be its true self.	1 (strongly disagree) to 5 (strongly agree)	Kanat-Maymon et al. (2021)
Daily receiving of need support	I feel that my pet really cares for and loves me. When I interact with my pet, it makes me feel competent. When I interact with my pet, it makes me feel free to be who I really am.	1 (strongly disagree) to 5 (strongly agree)	Kanat-Maymon et al. (2021)
Psychological distress	I tent to feel useless. I tend to feel depressed. I tend to feel worthless.	1 (strongly disagree) to 5 (strongly agree)	Dyer et al. (2014), (Kanat-Maymon et al. 2021)
Subjective well-being	Overall, I feel satisfied with my life. Overall, I feel happy. Overall, I feel positive about my life.	1 (strongly disagree) to 5 (strongly agree)	Diener (1984), Dyer et al. (2014), Kanat-Maymon et al. (2021)
Social isolation during Covid-19	How often did you feel that you lack companionship during the Covid-19 lockdown period? How often did you feel left out (of society) during the Covid-19 lockdown period? How often did you feel isolated during the Covid-19 lockdown period? How often did you feel lonely during the Covid-19 lockdown period?	1 (never) to 4 (often)	Hughes et al. (2004), Oliva and Johnston (2021)

Source: own illustration.

### The sample

Before collecting the data for our main study, we tested our items in two pretest rounds and refined the items accordingly. We then collected our data via MTurk, which we considered to be a useful tool for our data collection purposes, since it is frequently used in pet research (Bao and Schreer 2016; Brown et al. 2016; McConnell et al. 2019) and has become a well-accepted platform for collecting social sciences data (Lowry et al., 2016; Owens and Hawkins, 2019).

In October 2020, during the ongoing global Covid-19 pandemic, we requested a sample of 250 people who met the following quality criteria: (1) Being a citizen of the USA and (2) being an MTurk worker with a human intelligence task (HIT) approval rate of at least 95%. Of these 250 responses, we rejected those who indicated that they were citizens of another country and those with counterintuitive answers or straight-lined response patterns. We did not have to deal with many missing values, since most of our survey questions were mandatory. For the remaining missing values, we used the mean replacement procedure. Our final sample comprises 215 pet owners, which is considered sufficient compared to other studies’ sample sizes (Bao and Schreer 2016; Brown et al. 2016; Kanat-Maymon et al. 2021).

The descriptive statistics (Table 2) show that nearly two-thirds of the respondents are male (63.3%) and approximately one-third are female (36.7%). The majority are under the age of 35 years (61.4%). Just one-fifth (18.6%) of our respondents live alone, while more than one-third lives with a partner (37.2%) or a partner and children (29.8%). A total of 60% of the respondents live in urban areas, while the rest lives in suburban or rural areas. Most of the respondents are working full-time (84.7%). The average household income in our sample lies around the average of the USA (Guzman 2020). Approximately two-thirds of the respondents adopted at least one of their pets within sixth months prior to our data collection, that is during Covid-19-times (62.3%).

**Table 2:** Demographics

Sample criteria	N	%
Gender		
Male Female	136 79	63.3 36.7
Age		
Under 18 18–35 36–49 50–64 64 +	1 130 62 21 1	< 1.0 60.5 28.8 9.77 < 1.0
Living status		
Living alone Only living with a partner Only living with children Only living with older people Living with partner and children Multi-generational household	40 80 15 9 64 2	18.6 37.2 7.0 4.2 29.8 < 1.0
Place of living		
Urban Suburban Rural	129 63 22	60.0 29.3 10.2
Occupational status		
Full-time Part-time Self-employed Unemployed In education Retired Disabled	182 11 13 4 2 2 1	84.7 5.1 6.1 1.9 < 1.0 < 1.0 < 1.0
Income		
< 15,000 15,000–29,999 30,000–49,999 50,000–74,999 75,000–99,999 100,000–150,000 > 150,000	18 42 57 54 26 14 4	8.4 19.5 26.5 25.1 12.1 6.5 1.9
Newly adopted pets		
Adopted a pet within last six months Already owned pet	134 81	62.3 37.7

Source: own calculations.

We also asked specific Covid-19 questions (Table 3)in accordance with Oliva and Johnston (2021). Almost a third of the respondents had been diagnosed with Covid-19 (27.4%). While almost all of the respondents decided to minimize their social contacts (92.1%), almost half of them (49.3%) reported experiencing no significant changes in this regard. Approximately a third (34.4%) lost their employment due to Covid-19.

**Table 3:** Covid-19-specific data

Sample criteria	N	%
Diagnosed with Covid-19 Know someone diagnosed with Covid-19 Required to self-isolate/quarantine Chose to minimize social contacts Essential worker Lost employment due to Covid-19 No significant change experienced	59 120 135 198 104 74 106	27.4 55.8 62.8 92.1 48.4 34.4 49.3

Source: own calculations.

### Methodology

To test the relationships in our path model, we applied a variance-based statistical analysis method, namely PLS-SEM, which allows for analyzing the strength of the explanatory constructs’ influence on a target construct (Hair et al. 2022). This methodology’s main objective is the explanation and prediction of a target construct, hence, the constructs in the path model are arranged in a causal-predictive manner (Chin et al. 2020). PLS-SEM thereby supports the extension of existing theories as well as new theory development (Richter et al. 2016). PLS-SEM is particularly suitable when a complex model is tested for its predictive power (Hair et al. 2019). Research in a variety of social sciences disciplines has successfully applied PLS-SEM, such as ecological economics (Saari et al. 2021), higher education research (Ghasemy et al. 2021), sports marketing (Liu et al. 2021), and prior studies on happiness and satisfaction (Núñez-Barriopedro et al. 2020). Consequently, we opted for the PLS-SEM methodology and used the SmartPLS 3.3 software (Ringle et al. 2015) to assess our structural path model.

## Results and assessment

The measurement and structural model assessment follows the guidelines by Hair et al. (2022) and Hair et al. (2019). We use the bootstrapping procedure with 10,000 sub-samples and the two-tailed test based on a 95% confidence level for significance estimation. Reflective measurement models are assessed by analyzing the indicator reliability, internal consistency, convergent validity, and discriminant validity (Table 4). All our indicator loadings in the reflective measurement models are above the recommended threshold of 0.708.

**Table 4:** Indicator loadings

Construct	Item	Loading	CI
Human-pet relationship need support	Daily giving of need support. Daily receiving of need support.	0.915*** 0.946***	[0.873, 0.943] [0.923, 0.965]
Psychological distress	I tend to feel useless. I tend to feel depressed. I tend to feel worthless.	0.930*** 0.940*** 0.944***	[0.903, 0.950] [0.922, 0.954] [0.929, 0.957]
Social isolation during Covid-19	How often did you feel that you lacked companionship during the Covid-19 lockdown period? How often did you feel left out (of society) during the Covid-19 lockdown period? How often did you feel isolated during the Covid-19 lockdown period? How often did you feel lonely during the Covid-19 lockdown period?	0.895*** 0.848*** 0.820*** 0.873***	[0.866, 0.919] [0.804, 0.883] [0.745, 0.872] [0.832, 0.905]
Subjective well-being	Overall, I feel satisfied with my life. Overall, I feel happy. Overall, I feel positive about my life.	0.813*** 0.800*** 0.879***	[0.723, 0.875] [0.701, 0.861] [0.824, 0.916]

Source: own calculations. Note: *** = p < 0.01; CI = confidence interval.

We evaluate the constructs’ internal consistency reliability using ρ_A_ (Table 5). The values of the constructs human-pet relationship need support, social isolation during Covid-19 times, and subjective well-being are satisfactory, while the value of psychological distress is slightly higher, but still acceptable. We use the average variance extracted (AVE), which exceeds the threshold of 0.5 for all our constructs, to estimate the constructs’ convergent validity.

**Table 5:** Reliability and convergent validity

Construct	ρ_A_	AVE
Human-pet relationship need support	0.878	0.867
Psychological distress	0.933	0.879
Social isolation during Covid-19	0.893	0.738
Subjective well-being	0.787	0.691

Source: own calculations. Note: AVE = average variance extracted.

We use the heterotrait-monotrait (HTMT) criterion to determine discriminant validity (Henseler et al. 2015). Our values, including the upper bound of the confidence intervals, are all below the more conservative cut-off value of 0.85, and can therefore be clearly distinguished from one another (Table 6). Consequently, we assume discriminant validity.

**Table 6:** Discriminant validity/HTMT values

Constructs	Human-pet relationship need support	CI_0.90_	Psychological distress	CI_0.90_	Social Isolation during Covid-19	CI_0.90_
Psychological distress	0.363	[0.487]				
Social isolation during Covid-19	0.211	[0.342]	0.693	[0.768]		
Subjective well-being	0.448	[0.595]	0.284	[0.386]	0.108	[0.257]

Note: CI_0.90_ = upper bound of the confidence interval; one-tailed test; own calculations.

Next, we evaluate the structural model, which includes examining potential collinearity issues and testing the path coefficients’ significance and relevance. Our inner VIFs are all below the more conservative threshold of 3. Our highest value is 1.790, consequently, we assume that there are no critical collinearity issues. To evaluate the R² values (Table 7), we compare our results with the ones in previous research. Since other studies report similar R² values, for example, the model by Demir and Özdemir (2010), which explained 14% of the variance in happiness through friendship quality and need satisfaction, we assume that our R² values are within a good range. Furthermore, we test the predictive relevance using the PLS_predict_ method. We rely on the outline by Shmueli et al. (2019) to test for out-of-sample predictive power. Concurrently, we interpret the Q?_predict_ statistic and find that our Q?_predict_ values show predictive relevance (Table 7).

**Table 7:** R² and PLS_predict_

Construct	R²	Q²_predict_
Psychological distress	0.441	0.090
Social isolation during Covid-19	0.037	0.022
Subjective well-being	0.164	0.126

Source: own calculations.

We further find almost all of the path coefficients in our proposed model to be significant (Table 8). Human-pet relationship need support diminishes psychological distress (ß = − 0.207) and social isolation during Covid-19 times (ß = − 0.192), and, furthermore, has a positive effect on subjective well-being (ß = 0.329). Social isolation due to Covid-19 increases perceived psychological distress (ß = 0.593), while psychological distress decreases subjective well-being (ß = − 0.202). Only the path coefficient between social isolation during Covid-19 and subjective well-being is not significant. Following Geerling and Diener (2020), we also consider the strength of our path coefficients. We find the largest effect between social isolation due to Covid-19 and psychological distress, while human-pet relationship need support is the second largest in its terms. The paths running from human-pet relationship need support to psychological distress as well as to social isolation due to Covid-19, and the one from psychological distress to subjective well-being, are almost equal in size.

**Table 8:** Path coefficients

Path	ß
Human-pet relationship need support → Subjective well-being	0.329***
Human-pet relationship need support → Psychological distress	− 0.207***
Human-pet relationship need support → Social isolation due to Covid-19	− 0.192***
Social isolation due to Covid-19 → Psychological distress	0.593***
Psychological distress → Subjective well-being	− 0.202**
Social isolation due to Covid-19 → Subjective well-being	0.100

Source: own calculations. Note: *** = p < 0.01, ** = p < 0.05.

According to our mediation analysis, only one of our mediators, namely psychological distress, shows a significant indirect effect. Owing to social isolation’s insignificant direct effect, this construct does not serve well as a mediator between human-pet relationship need support and subjective well-being. Furthermore, we do not find empirical support for the sequential mediation. The specific indirect effects are shown in Table 9 and the total effects are shown in Table 10. To summarize, we find that psychological distress has a partial mediating effect on the relationship between human-pet relationship need support and subjective well-being.

**Table 9:** Specific indirect effects

Path	ß
Human-pet relationship need support → Psychological distress → Subjective well-being	0.042*
Human-pet relationship need support → Social isolation due to Covid-19 → Subjective well-being	− 0.019
Human-pet relationship need support → Social isolation due to Covid-19 → Psychological distress → Subjective well-being	0.023

Source: own calculations. Note: * = p < 0.1.

**Table 10:** Total effects

Path	ß
Human-pet relationship need support → Subjective well-being	0.375***
Human-pet relationship need support → Psychological distress	− 0.321***
Human-pet relationship need support → Social isolation due to Covid-19	− 0.192***
Social isolation due to Covid-19 → Psychological distress	0.593***
Psychological distress → Subjective well-being	− 0.202**
Social isolation due to Covid-19 → Subjective well-being	− 0.020

Source: own calculations. Note: *** = p < 0.01, ** = p < 0.05.

## Discussion and implications

We adopted the Kanat-Maymon et al. (2016) and Kanat-Maymon et al. (2021) models for our study, and adapted them in various ways for the following reasons. First, we excluded closeness to pets from our data analysis because the construct did not show enough discriminant validity in relation to human-pet relationship need support, meaning that these constructs are not clearly distinguishable from one another. Second, we used giving and receiving need support as a HOC, since we assume both LOCs to be facets of basic human needs rather than being independent constructs. Third, we used distress as a precursor of subjective well-being instead of a separate target construct by assuming that psychological distress has an effect on subjective well-being (Meng and D’Arcy 2016). Fourth, we adapted subjective well-being’s measurement. In keeping with the definition by Diener (1984), we define subjective well-being according to the three dimensions happiness, life-satisfaction, and positive affect, which have been proven to be a close operationalization of subjective well-being.

Our findings confirm five out of six hypotheses (H1 to H5). In the human-pet relationship, giving and receiving need support not only decrease psychological distress and the feeling of being socially isolated due to Covid-19, but simultaneously increase the pet owner’s overall subjective well-being. According to our findings, psychological distress decreases subjective well-being, while the feeling of being socially isolated during Covid-19 times increases psychological distress in the human-pet relationship, but does not decrease subjective well-being. Therefore, surprisingly, hypothesis H6 could not be confirmed. Reasons for this finding might be that (1) the Covid-19 pandemic did not generally influence our respondents’ overall subjective well-being to any great extent; (2) since subjective well-being is more long-term oriented, this effect might not have occurred, yet, but could in the future; or (3) social isolation arouses stress, which makes people generally feel less happy than usual, thereby influencing subjective well-being. The finding that social isolation due to Covid-19 increases psychological distress, which decreases subjective well-being is alarming, since it remains unclear when the pandemic, and, consequently, social isolation, will end.

With regard to *theoretical implications*, prior empirical studies of the pets’ effect on human health and well-being have produced conflicting findings (Fraser et al. 2020). In our study, the need support derived from the relationship with one’s pet decreases one’s perceived psychological distress and the effect of social isolation due to Covid-19, as well as having a positive effect on subjective well-being. Consequently, our findings contradict those of Gilbey et al. (2007) who found that the acquisition of a companion animal does not alleviate loneliness. Nevertheless, our findings might differ because we did not measure loneliness directly, but perceived social isolation during Covid-19 times, which is a rather special and under-researched situation.

Our model has *practical implications* on three levels. First, on the individual level, we find that having a pet as a companion animal has positive implications for one’s subjective well-being and decreases distress during times of social isolation. One should, of course, keep in mind that pets are not a toy—adopting a pet should be a well-thought-out decision at any time (Oliva and Johnston 2021). Second, on an industry level, pet owners allow the pet industry to make large profits, which, in turn, benefit the economy. Third, on a societal level, our model informs social policy makers, since pet companionship might lower the chances of people suffering from psychological illnesses, due to decreased psychological distress, thereby decreasing the costs of treatment and the health care system as a whole. Even a small increase in pet-related health improvements can result in large savings within the health care system (Headey 1999), due to healthy humans needing fewer doctoral appointments, which saves costs for the health care system and society as a whole.

Since psychological needs are considered to universally induce growth rather than being culture or personality-specific (Rodríguez-Meirinhos et al. 2020), happiness is an important research topic in several cultures (Delle Fave and Bassi 2009), and its core definition is stable across countries(Delle Fave et al. 2016). Consequently, our findings’ implications are likely to not only apply to USA citizens, but also to a wider community. Policy makers could, therefore, encourage people to become or remain pet owners by, for example, allowing them to take companionship dogs to work (Hall and Mills 2019; Hall et al. 2017). Wells and Perrine (2001) found that employees who bring their pets to their workplace perceive lower stress levels and enjoy other health benefits, thereby also benefiting the organization.

However, people might not equally receive as much benefit from the human-pet relationship as indicated in our study. Our findings hold as long as the pet owner’s psychological needs are met and they perceive this relationship as a resource (according to the COR). Therefore, we need to focus on how the human-pet relationship can be enhanced, so that humans as well as pets can derive as much benefit from it as possible. A variety of studies have shown the relevance of oxytocin in mammal social behavior and emotional regulation (Kis et al. 2014; Pedretti et al. 2021; Somppi et al. 2017). In taking the human-dog interaction and communication as an example, dogs learn from the interaction with humans (Barrera et al. 2018), so pet owners should frequently interact with their dog and provide them learning opportunities for enhanced communication. Not only dogs need to learn how to interact with humans, but also humans need to learn how to properly interact with dogs. For instance, dogs tend to respond better to gestures than verbal interaction (D’Aniello et al. 2016) or visual contact (Ittyerah and Gaunet 2009). Nevertheless, when visual contact is pursued by the dog, they mostly look the human in the eye instead of reading facial expressions (Kis et al. 2017). Knowing this, we can more effectively and efficiently interact with our pet.

## Conclusion and future research

The Covid-19 pandemic has forced the global community to cope with isolation, while at the same time strengthening the bond between humans and their pets (Mayers 2021). Our model explains and predicts the human-pet relationship need support effect on subjective well-being by adding psychological distress and social isolation during Covid-19 times as mediators. We find that human-pet relationship need support increases subjective well-being, and decreases psychological distress, as well as loneliness, which is caused by social isolation during Covid-19 times. Furthermore, psychological distress decreases subjective well-being, whereas perceived loneliness due to social isolation during Covid-19 does not. We conclude that engaging in a relationship with a pet can help humans decrease their levels of perceived loneliness and stress in everyday life—especially in times of crises. Furthermore, Cummins et al. (2003) found that after experiencing a negative event, the level of a person’s well-being will return to its old “normal”. Until then, the companionship of an animal might bridge this hardness and, later on, add happiness to one’s life in better times, since the Covid-19 pandemic can be seen as a negative event to one’s life.

Our study has several limitations and provides avenues for further research. First, we solely collected our data in the USA and used self-reported measures, which could be prone to common method bias. Moreover, only about one-fifth of the respondents indicated that they lived alone. Consequently, the effects might be higher among those without social contact at home. In addition, we did not differentiate between the type of pet under consideration. Future studies might look at the difference in the capacity of the human-relationship to fulfill psychological needs of owners and its consequences depending on the type of companion animal. As such, this study lays a foundation for future research on the benefits of human-pet relationships in times of crises. In general, a broader sample is needed to further test our model and substantiate our findings across gender, age, culture, and pets to further explore potential differences. This is an important avenue for future research, since Miller et al. (1992) found that positive effects from pets are perceived differently by females and males. Zasloff (1996), among others, noted that even though humans gain benefits from several companion animal relationships, these are not necessarily the same.

Second, we used a cross-sectional design, which has its known limitations. Therefore, other data collection approaches could be used in future research, such as longitudinal designs, which allow for comparisons across time, that is during and after crises. Even though we use a causal-predictive methodology, statements about causality cannot be drawn with certainty. Though there are studies, which have indicated that the relationship between pet ownership and health conditions might be causal and not only correlational (Headey and Grabka 2007). Nevertheless, these Covid-19 days have been tough times for all of us, and our study shows how companion animals can help us live a happier and more balanced life, as well as potentially and presumably vice versa.

## Data Availability

The authors used the software SmartPLS 3.3 for their data analysis.
